# hTERT protein expression is independent of clinicopathological parameters and c-Myc protein expression in human breast cancer

**DOI:** 10.1186/1477-3163-4-17

**Published:** 2005-10-04

**Authors:** AE Elkak, G Meligonis, M Salhab, B Mitchell, JRS Blake, RF Newbold, K Mokbel

**Affiliations:** 1 The Breast Unit, St George's Hospital, Blackshaw Road, London, SW17 0QT, UK; 2 Department of Histopathology, King's Mill Hospital, Mansfield, UK; 3 Department of Surgery, King's Mill Hospital, Mansfield, UK; 4 Institute of Cancer Genetics and Pharmacogenomics, Brunel University, Uxbridge, Middlesex. UB8 3PH, UK

## Abstract

**Background:**

Telomerase is a ribonucleoprotein enzyme that synthesises telomeres after cell division and maintains chromosomal length and stability thus leading to cellular immortalisation. The hTERT (human telomerase reverse transcriptase) subunit seems to be the rate-limiting determinant of telomerase and knowledge of factors controlling hTERT transcription may be useful in therapeutic strategies. The hTERT promoter contains binding sites for c-Myc and there is some experimental and in vitro evidence that c-Myc may increase hTERT expression. We previously reported no correlation between c-Myc mRNA expression and hTERT mRNA or telomerase activity in human breast cancer. This study aims to examine the correlation between hTERT expression as determined by immunohistochemistry and c-Myc expression, lymph node status, and tumour size and grade in human breast cancer.

**Materials and methods:**

The immunohistochemical expression of hTERT and c-Myc was investigated in 38 malignant breast tumours. The expression of hTERT was then correlated with the lymph node status, c-Myc expression and other clinicopathological parameters of the tumours.

**Results:**

hTERT expression was positive in 27 (71%) of the 38 tumours. 15 (79%) of 19 node positive tumours were hTERT positive compared with 11 (63%) of 19 node negative tumours. The expression was higher in node positive tumours but this failed to reach statistical significance (p = 0.388). There was no significant association with tumour size, tumour grade or c-Myc expression. However, hTERT expression correlated positively with patients' age (correlation coefficient = 0.415, p = 0.0097).

**Conclusion:**

hTERT protein expression is independent of lymph node status, tumour size and grade and c-Myc protein expression in human breast cancer

## Introduction

Telomeres are highly specialised structures at the end of each chromosome which contain tandem repeat DNA sequences? In humans, this sequence is TTAGGG [1]. According to the telomere/telomerase hypothesis, the telomeric ends of chromosomes of normal somatic cells replicate inefficiently and progressively shorten at each cell division until cumulative loss impairs vital cellular functions and the cells exit the cell cycle and undergo apoptosis [2].

Telomerase is a ribonucleoprotein enzyme that contains an RNA template complementary to the (TTAGGG) n repeats and thus synthesises telomeres after cell division and maintains chromosomal length and stability leading to immortalisation [3-6]. Accordingly, telomerase activity has been investigated and detected in a wide range of human malignancies, germline cells and immortal cells but not in normal somatic cells [3-8]. Human telomerase consists of an RNA subunit – human telomerase RNA (hTR) [9], a protein component (human telomerase associated protein 1 – hTEP1) [10] and the catalytic subunit hTERT (human telomerase reverse transcriptase) [11-13]. Of these subunits telomerase activity requires the presence of hTR, which is the RNA template for the telomeric repeat, and hTERT, which is the reverse transcriptase. The gene coding for hTERT has been cloned and mapped to 5p15.33 [14]. hTERT is a 127-kDa protein whose function closely correlates with telomerase activity [10,15,16].

Investigation of the mechanisms of hTERT control is important in elucidating the pathways that may be amenable to therapeutic manipulation and one such pathway involves the transcription factor Myc.

An increased level of c-Myc occurs frequently in a wide range of tumours [17-23] due to de-regulated expression of myc through gene amplification, retroviral insertion or chromosomal translocation. Sequence analysis of the hTERT gene promoter has shown the presence of at least 2 and perhaps as many as 29 E boxes [24], potential binding sites for the Myc oncoprotein, and the possibility of a regulatory role for Myc has been explored in a number of studies. It has been found that purified Myc interacts with the E box sequences and that cotransfection of Myc induces activity in the isolated hTERT promoter [25]. It has been shown that retroviral expression of c-myc increases the amount of hTERT mRNA in human mammary epithelial cells and fibroblasts and telomerase activity could thereafter be detected [26]. It has also been reported that expression of c-Myc leads to increased hTERT expression and telomerase activity in human B cells [27]. Moreover, since this does not require protein synthesis this appears to be due to a direct effect of Myc on the hTERT promoter and not secondary to an increase in cellular proliferation by Myc [27]. In addition, the introduction of Myc anti-sense RNA has been shown to lead to a reduction in hTERT promoter activity [25]. Latil et al [28] demonstrated a relationship between hTERT and c-Myc expressions in prostate cancer whilst other investigators [29] have found that Mad1, another member of Myc pathway, is a suppressor of hTERT at the level of transcription.

Using RT-PCR, we found that hTERT mRNA expression was significantly higher in breast cancer tissues compared with non-cancerous breast tissues [30]. However, we did not observe any relationship between hTERT expression and tumour stage, patients' age or c-Myc expression. Due to the potential limitations of mRNA studies which we highlighted in our previous report, we planned to determine the protein expression of both hTERT and c-Myc using immunohistochemistry (IHC) and investigate any potential associations.

The aim of this study was to determine the (IHC) expression of hTERT in human breast cancer and to examine the association between hTERT expression and clinicopathological parameters of the tumours (size, grade, and nodal status) and c-Myc expression.

## Methods

Institutional guidelines including ethical approval were followed. Patients were treated with wide local excision or mastectomy and axillary node dissection. Patients with estrogen and /or progesterone positive tumours received tamoxifen. Radiotherapy was administered to all patients who had breast conserving surgery and chemotherapy to patients with lymph node positive or high grade tumours. Using IHC, the expressions of hTERT and c-Myc were determined in 38 breast cancer specimens (19 specimens were lymph node positive tumours and 19 were lymph node negative tumours the specimens).

### Immunohistochemistry

#### Staining

Paraffin sections were dewaxed in xylene (two changes each for 5 minutes). Endogenous peroxidase was blocked in 480 ml of Methanol and 6 ml of H2O2 for 15 minutes and rinsed in running tap water for 5 minutes. Heat mediated antigen retrival was performed as follows: 1 litre of pH6 Citrate buffer was placed in the microwave into the pressure cooker, then heated on full power for 10 minutes.

Sections were placed into the boiling buffer. Pressure cooker was heated on full power until the pressure is attained and then cooked for a further 2 minutes. Sections were then rinsed well in running tap water and placed in Tris Buffer. Primary antibody preparations were applied and incubated for 45 minutes then washed in TRIS Buffer (pH 7.6). The antibodies used are: NCLL-hTERT (Clone 44F12, 1:50 dilution, Novocastra Laboratories Ltd, Newcastle upon Tyne, UK) and NCL-cMYC (Clone 9E11, 1:200 dilution, Novocastra Laboratories Ltd, Newcastle-upon-Tyne, UK). 5% goat serum was used for dilution. Super enhancer was then applied for 20 minutes. Sections were washed in TRIS Buffer (pH 7.6). Poly-HRP was then applied for 30 minutes. Sections were washed in TRIS Buffer (pH 7.6). DAB solution was then applied for 10 minutes. Slides were washed in distilled water, bleached to de-activate the DAB and rinsed in running tap water. Sections were then counterstained with Harris Haematoxylin.

### Evaluation of immunohistochemical staining

Two observers assessed the sections using the following criteria:

#### hTERT

**Figure 1:**
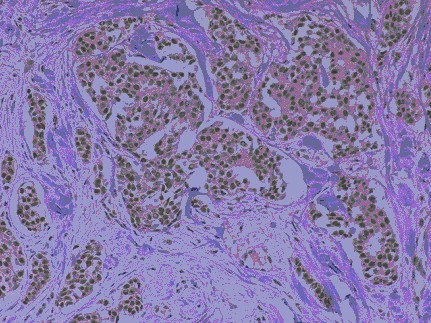
Invasive ductal carcinoma – hTERT positive: 2 (strong nuclear staining).

**Figure 2:**
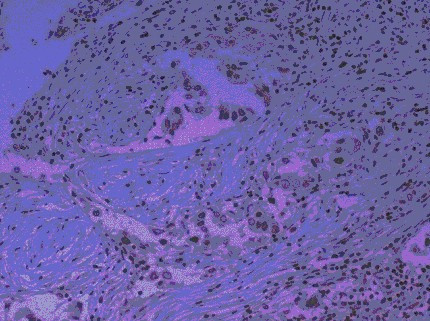
Invasive ductal carcinoma – hTERT positive: 1 (moderate nuclear staining of tumour cells and strong staining of surrounding lymphocytes.

Scores were assigned as follows: 2, strong staining throughout nucleus (Fig 1); 1, moderate staining of nucleus or dotted staining of nucleolus (Fig 2); 0, no staining.

#### c-Myc

**Figure 3:**
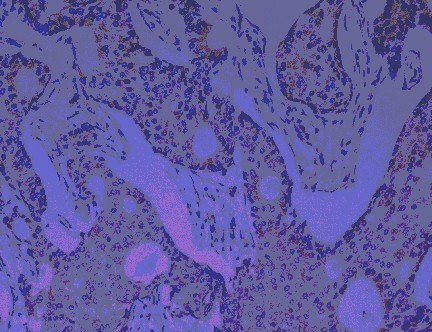
Invasive ductal carcinoma – c-Myc positive: 2 (moderate cytoplasmic staining).

**Figure 4:**
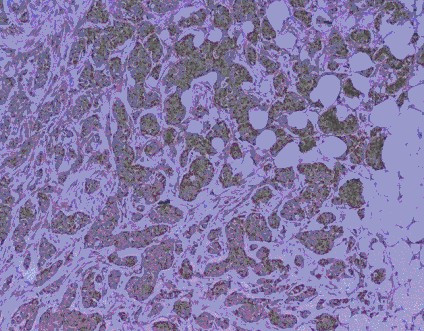
Invasive ductal carcinoma – c-Myc positive: 3 (strong cytoplasmic staining).

Cytoplasmic staining intensity was graded as: no staining (0), weak (1), moderate (2) (Fig 3), or strong (3) (Fig 4). The percentage of tumour cells with c-Myc staining was scored as follows: 1, <5%; 2, 5–20%; 3, 21–50%; 4, >50%. Then the multiplication values were grouped into four scores as 0, (multiplication values 0, 1); 1, (multiplication values 2, 3); 2, (multiplication values 4, 6); or 3, (multiplication values 8, 9, 12).

### Statistical analysis

Chi Square test was used to study the relationship between hTERT expression in lymph node positive and lymph node negative tumours. The expression of hTERT in tumours was then correlated with c-Myc expression, clinicopathological parameters of the tumours (size and grade) and patients' age. A p-value of < 0.05 was considered statistically significant.

## Results

hTERT expression was positive in 27 (71%) of 38 tumours. 15 (79%) of the 19 node positive tumours were hTERT positive compared with 11 (63%) of the node negative tumours. Although hTERT expression was higher in node positive tumours (median score 1.0 Vs 0), however this failed to reach statistical significance (p = 0.388). There was no significant correlation with tumour size, tumour grade, hormone receptor status or c-Myc expression. Interestingly, hTERT expression correlated positively with patients' age (correlation coefficient = 0.415, p = 0.0097). Table 1 demonstrates hTERT and c-Myc expression and the clinicopathological characteristics of the tumours studied.

**Table 1:** hTERT and c-Myc expression and the clinicopathological characteristics of the tumours

**No**	**hTERT**	**Int**	**%**	**Multip**	**Final**	**Hist**	**Age**	**Size**	**Grade**	**LN**	**ER**
1	1	3	4	12	3	D	77	23	2	P	P
2	1	1	1	1	0	D	60	13	3	P	P
3	2	2	4	8	3	D	75	28	3	P	P
4	2	3	4	12	3	D	68	35	3	P	P
5	1	3	4	12	3	D	63	12	2	P	P
6	1	2	4	8	3	D	74	14	3	P	P
7	1	3	4	12	3	D	48	28	2	P	P
8	2	2	4	8	3	D	72	50	3	P	N
9	2	3	4	12	3	D	58	30	2	P	P
10	0	3	4	12	3	L	50	35	2	P	P
11	1	3	3	9	3	D	38	30	3	P	N
12	1	2	4	8	3	D	56	20	3	P	N
13	1	1	4	4	2	D	54	50	2	P	P
14	0	3	4	12	3	L	43	20	1	P	P
15	0	3	4	12	3	L	43	20	1	P	P
16	0	2	4	8	3	DL	48	23	2	P	P
17	1	3	4	12	3	DL	82	35	2	P	P
18	1	3	4	12	3	PL	73	50	3	P	P
19	1	1	4	4	2	D	50	18	2	P	P
20	1	3	4	12	3	DL	61	6	2	N	P
21	1	3	4	12	3	D	59	15	2	N	P
22	1	3	4	12	3	D	63	26	3	N	P
23	0	2	4	8	3	P	66	13	2	N	P
24	1	3	4	12	3	D	61	15	3	N	P
25	1	3	4	12	3	D	65	6	1	N	P
26	2	3	4	12	3	D	51	35	3	N	P
27	1	3	4	12	3	D	50	12	2	N	P
28	2	2	4	8	3	TL	70	12	1	N	P
29	1	3	4	12	3	TL	62	19	2	N	P
30	0	3	4	12	3	T	43	8	1	N	P
31	2	2	4	8	3	M	54	14	1	N	P
32	1	3	4	12	3	D	60	13	1	N	P
33	1	3	4	12	3	A	56	11	2	N	N
34	0	3	4	12	3	D	57	10	2	N	P
35	0	3	4	12	3	D	53	18	2	N	P
36	0	2	4	8	3	D	55	10	2	N	P
37	0	2	4	8	3	D	52	20	3	N	P
38	0	1	2	2	1	D	64	12	3	N	N

## Discussion

Our observation that hTERT protein is expressed in most breast tumours is expected and consistent with our previous reports using mRNA and enzyme measurements [30-32]. The lack of is correlation between hTERT protein expression and tumour size, grade or nodal status is also consistent with our previous study using mRNA and RT-PCR technology [30]. However we previously reported that telomerase activity correlated with these clinicopathological parameters [31,32]. Although hTERT expression is associated with malignancy, it does not seem to correlate with tumour stage. This is probably a true observation as it has been demonstrated using both RT- PCR and immunohistochemistry and could be explained on the basis of post-transcriptional modification.

Bieche et al [33] reported a positive correlation between hTERT and c-Myc gene expression. Furthermore, other investigators [34-36] demonstrated that hTERT gene is a direct target of c-Myc. Although the hTERT promoter contains E-boxes, consistent with the findings of the present study, we previously observed no correlation between c-Myc mRNA levels and telomerase activity [37] and no association between hTERT and c-Myc at the mRNA level [30]. The control of hTERT is undoubtedly a complex one and it is likely that a number of other transcription factors influence its expression than c-Myc. These might act together with c-Myc, as has been shown for Sp1 [38] or independently. In this respect, it has been shown that transfer of a normal chromosome 3 into human breast carcinoma cells results in abolition of hTERT transcripts without any change in c-Myc levels [39]. Furthermore, it is known that another member of the Myc family, Mad1 forms a complex with Max and acts as a transcriptional repressor at the same binding sites as Myc-Max. It has been shown that, the proportion of Mad1 binding to the hTERT promoter rises and that of Myc falls, during the differentiation of HL60 cells [37,40]. This is associated with reduced acetylation of the hTERT promoter and measurement of the Mad/Myc ratio is likely to be important in establishing the overall level of transcriptional activation of hTERT.

## Abbreviations

Int: c-Myc intensity

%: c-Myc percentage positive

Multip: c-Myc multiplication

Final: Final c-Myc score

Hist: Histology

D: ductal

L: Lobular

DL: Ductal and Lobular

P: Papillary

M: Mucoid

A: Apocrine

T: Tubular

LN: Lymph node status, P: positive, N: negative

ER: Oestrogen receptor status, P: positive, N: negative
